# Transmission of *Streptococcus equi* Subspecies *zooepidemicus* Infection from Horses to Humans 

**DOI:** 10.3201/eid1907.121365

**Published:** 2013-07

**Authors:** Sinikka Pelkonen, Susanne B. Lindahl, Päivi Suomala, Jari Karhukorpi, Sakari Vuorinen, Irma Koivula, Tia Väisänen, Jaana Pentikäinen, Tiina Autio, Tamara Tuuminen

**Affiliations:** Finnish Food Safety Authority Evira, Kuopio, Finland (S. Pelkonen, T. Autio);; National Veterinary Institute, Uppsala, Sweden (S.B. Lindahl),;; Swedish University of Agricultural Sciences, Uppsala (S.B. Lindahl);; Eastern Finland Laboratory Centre Joint Authority Enterprise, Mikkeli, Finland (P. Suomala, T. Tuuminen);; Eastern Finland Laboratory Centre Joint Authority Enterprise, Joensuu, Finland (J. Karhukorpi);; Eastern Finland Laboratory Centre Joint Authority Enterprise, Kuopio (J. Pentikäinen); Mikkeli Central Hospital, Mikkeli (S. Vuorinen);; Kuopio University Hospital, Kuopio (I. Koivula, T. Väisänen); University of Helsinki, Helsinki, Finland (T. Tuuminen)

**Keywords:** zoonoses, β-hemolytic streptococcus, Streptococcus equi subsp. zooepidemicus, sepsis, septic arthritis, meningitis, abscess, equine, bacteria, zoonoses, streptococci

## Abstract

*Streptococcus equi* subspecies *zooepidemicus* (*S. zooepidemicus*) is a zoonotic pathogen for persons in contact with horses. In horses, *S. zooepidemicus* is an opportunistic pathogen, but human infections associated with *S. zooepidemicus* are often severe. Within 6 months in 2011, 3 unrelated cases of severe, disseminated *S. zooepidemicus* infection occurred in men working with horses in eastern Finland. To clarify the pathogen’s epidemiology, we describe the clinical features of the infection in 3 patients and compare the *S. zooepidemicus* isolates from the human cases with *S. zooepidemicus* isolates from horses. The isolates were analyzed by using pulsed-field gel electrophoresis, multilocus sequence typing, and sequencing of the *szP* gene. Molecular typing methods showed that human and equine isolates were identical or closely related. These results emphasize that *S. zooepidemicus* transmitted from horses can lead to severe infections in humans. As leisure and professional equine sports continue to grow, this infection should be recognized as an emerging zoonosis.

*Streptococcus equi* subspecies *zooepidemicus* (*S. zooepidemicus*) is a β-hemolytic, Lancefield group C streptococcal bacterium. *S. zooepidemicus* is considered an opportunistic commensal in horses (*1*–*3*), but it may also cause infections in other domestic animals such as cattle, sheep, goats, pigs, dogs, and cats (*4*–*10*). Another subspecies of the same genus, *Streptococcus equi* subsp. *equi* (*S. equi*), causes strangles, the highly contagious and serious disease in horses (*1*,*11*,*12*).

*S. zooepidemicus* shares >98% DNA sequence homology with *S. equi* (*1*) and >80% DNA sequence homology with *Streptococcus pyogenes* (*13*), a Lancefield group A streptococcus and major human pathogen. Although considered an opportunistic pathogen, *S. zooepidemicus* shares important virulence factors with both *S. equi* and *S. pyogenes* such as the M-like proteins, superantigens (sAgs), and the presence of a hyaluronic acid capsule in certain strains. The variable M-protein, located on the surface of *S. pyogenes*, can be used to differentiate *S. pyogenes* strains serologically where the M1 serotype is associated with invasive disease in humans (*14*). The M-like, cell-wall–anchored surface protein SzP, found in all strains of *S. zooepidemicus*, is essential for the pathogenesis of the disease, at least in horses, where it binds fibrinogen and exhibits antiphagocytic activity that impairs with host protection. The sAgs SeeH, SeeI, SeeL, and SeeM found in *S. equi* share 96%–99% amino acid sequence homology with *S. pyogenes* sAgs SpeH, SpeI, SpeL, and SpeM (*15*). However, few investigated strains of *S. zooepidemicus* contain homologs to these sAgs. Instead, novel sAgs (SzeF, SzeN, and SzeP) have been identified in certain strains, sharing 34%–59% homology with SpeH, SpeM, and SpeL of *S. pyogenes* (*16*).

*S. zooepidemicus* has seldom been isolated from humans. Surprisingly, most published data on humans go back to the latter part of the 1980s (*17*). Occasional human infection was reported as a result of the consumption of homemade cheese or unpasteurized milk from cows with mastitis (*17*). In humans, *S. zooepidemicus* may cause glomerulonephritis and rheumatic fever, which are known sequelae of *S. pyogenes* (group A) infections (*18*). Meningitis and purulent arthritis have also been reported (*19*,*20*).

*S. zooepidemicus* displays a wide genetic variation between different isolates (*13*,*21*–*23*). The sequence of the SzP protein gene (*szP*) has been shown to vary greatly between different strains of *S. zooepidemicus* (*24*–*26*), and the variable regions of *szP* can be used to genetically differentiate strains within the subspecies (*27*–*29*). Pulsed-field gel electrophoresis (PFGE) is a DNA-based typing technique that is highly discriminatory and has been used in epidemiologic investigations of *S. zooepidemicus* outbreaks (*30*,*31*). 

Multilocus sequence typing (MLST) is a method for characterization of bacterial isolates by comparing sequences of several gene fragments. Webb et al. (*22*) developed a MLST protocol for *S. zooepidemicus* consisting of 7 housekeeping genes. Obtained sequences are compared to previously deposited sequences, and a sequence type (ST) is assigned from the online PubMLST *S. zooepidemicus* database (http://pubmlst.org/szooepidemicus) developed by Jolley et al. (*32*).

Within a 6-month period, through our routine practice, we found 3 cases of severe disseminated disease in humans caused by *S. zooepidemicus*. The purpose of this study was to 1) characterize the clinical presentation of the disease caused by *S. zooepidemicus*, 2) microbiologically characterize the isolated strains, and 3) identify clonality of human isolates for comparison to equine isolates from contact horse stables and other horse farms of the surrounding area. 

## Patient 1

A 57-year-old man, a farmer and horse breeder from central Finland, was admitted unconscious and febrile to the emergency room of a principal hospital in February of 2011. Cerebrospinal fluid (CSF) was collected, and he was referred to the intensive care unit. He had aortic valve insufficiency and had been catheterized 3 months earlier. His condition was septic, with clinical symptoms of meningismus and pulmonary congestion. The C-reactive protein (CRP) level was 564 mg/L (reference <3 mg/L) and the leukocyte count 15.9 × 10^9^ cells/L (reference 3.4–8.2 × 10^9^ cells/L). Microscopy staining of the CSF revealed gram-positive cocci in chains with a considerable number of polymorphonuclear cells. The next day, bacteria subsequently identified as *S. zooepidemicus* (Table 1) grew from the CSF and 4 of the 4 blood culture bottles, leading to a primary diagnosis of meningitis and sepsis. Intravenous high-dose penicillin treatment (5 weeks) was started in combination with gentamicin (first 10 days). Two and a half days after admission, the patient regained consciousness. Intravascular coagulopathy developed, and 20 days later, progressive endocarditis. The bicuspid native aortic valve was resected the same day, and several bacterial patches were observed. His perioperative blood cultures remained negative. Neurologic sequelae did not develop, but his recovery and rehabilitation required several weeks. 

**Table 1:** Bacteriologic identification of *Streptococcus equi* subspecies *zooepidemicus* isolates from patient samples in different ISLAB laboratories*

Isolate (patient, source)	APIStrep†	STR Rapid ID32†	Agglutination with group serum specimens	VITEK2 GP-ID†	AccuProbe
Hum1 (patient 1, CSF)	0063607 (99.9% *S. zooepidemicus)*	15412061151 (99.9% *S.* *zooepidemicus)*	Group C	Not done	Not done
Hum2 (patient 2, blood)	Not done	15412061151 (99.9% *S. zooepidemicus)*	Group C	053450364317451 (99% *S.* *zooepidemicus)*	*Streptococcus agalactiae‡*
Hum3 (patient 3, abdominal aortic wall)	Not done	15512061111 (99.9% *S. zooepidemicus)*	Group C	Not done	Not done

*ISLAB, Eastern Finland Laboratory Centre Joint authority Enterprise; CSF, cerebral spinal fluid. †bioMérieux, Marcy l’Etoile, France.  ‡*S. agalactiae* (315,588 reflective light units, the reference range below 50,000 reflective light units*). S. agalactiae* and *S. zooepidemicus* are known to cross-react in the AccuProbe Group B Streptococcus Culture ID Test (Gen-Probe, San Diego, CA, USA).

## Patient 2

A 62-year-old-man, a truck driver and horse trainer from eastern Finland, returned home from work in a febrile and confused state in May 2011. The next day, on hospital admission, he had pain and swelling of the right knee and right shoulder. He was hyperglycemic and had untreated non–insulin-dependent diabetes mellitus. The synovial fluid aspirated from his knee was turbid, with a leukocyte count of 86.0 × 10^9^ cells/L and a high percentage of polymorphonuclear cells (87%). The CRP level was 329 mg/L and the blood leukocyte count was 19.3 × 10^9^ cells/L. Antimicrobial drug therapy with intravenous cefuroxime was started. The next day, bacteria subsequently identified as *S. zooepidemicus* were cultured from his right knee and 4 of 4 blood culture bottles (Table 1). On the third day, arthroscopic synovectomy and irrigation of the right knee was performed, and the procedure was repeated. Cefuroxime was changed to intravenous vancomycin without therapeutic response. The CRP level remained high (229 mg/L) and the leukocyte count was 15.3 × 10^9^ cells/L. Next, a combination of penicillin G with clindamycin was administered. He had no evidence of endocarditis, but Tc99m scintigraphy revealed an uptake in the patient’s right shoulder and lower jaw region. Arthroscopic debridement and irrigation of the right shoulder were performed, and purulent synovial fluid was collected for culture. Antimicrobial drug therapy continued with intravenous cefuroxime and clindamycin for 2 weeks; thereafter, with oral cephalexin and clindamycin for 1 week. The patient’s clinical condition gradually improved, and finally, he was able to walk with crutches. He was discharged from the hospital 6 weeks after the onset of illness.

## Patient 3

A 49-year-old man, a horse trainer from eastern Finland, was admitted to the hospital in August 2011 because of severe, prolonged low-back pain. A horse had kicked his forehead 2–3 weeks earlier. The accident did not require medical attention at that time; however, the low-back pain had increased gradually. He had medicated himself with ibuprofen, 400 mg up to 20 tablets per day, without relief, except when lying supine. He did not record his temperature but was sweating after taking ibuprofen and sought medical attention when walking became difficult. His condition was treated as muscle pain. After a week he returned to the medical center because of excruciating pain in his back. There were no abnormal radiologic findings. On clinical examination, he was nonfebrile and had no clinical symptoms or hemodynamic abnormalities. The clinical findings were unremarkable, except for the pain in his lower back on percussion and a pulsating abdominal mass. Laboratory tests showed leukocytosis (16.2 × 10^9^ cells/L), an elevated erythrocyte sedimentation rate of 73 mm/h (reference 1–15 mm/h), and an elevated CRP level of 217 mg/L. Computed tomography revealed a psoas abscess (65 × 35 × 30 mm) linked to an infected aortic aneurysm (diameter 40 mm). The aneurysm was resected and replaced by a Y-prosthesis, and the psoas abscess was drained. Gram stain of tissue obtained through operation on the abdominal aorta and debridement of the psoas abscess revealed gram-positive cocci in 3 (2 from the aortic wall and 1 from the psoas abscess) of the 4 samples. The patient’s condition was treated with piperacillin-tazobactam, later replaced with intravenous penicillin. Transesophageal echocardiography showed no signs of endocarditis. The patient recovered without sequelae.

## Materials and Methods

### Microbiological Diagnostics of *S. zooepidemicus* Strains in Clinical Laboratories

Each clinical laboratory used the standard operating procedures and standard culture media of their own. CSF and synovial fluid samples were cultured on blood and/or chocolate agar and blood samples in blood culture bottles and incubated aerobically and anaerobically. For identification, Gram stain and agglutination with streptococcal group sera (Streptococcal Grouping Kit; Oxoid Ltd., Basingstoke, UK) were carried out in all laboratories. The identification of *S. zooepidemicus* to the species level varied between the laboratories, and was performed using at least one of the following tests as shown in Table 1: APIStrep, , STR Rapid ID32, or VITEK2 GP-ID (all from bioMérieux Marcy l’Etoile, France), combined with AccuProbe Group B Streptococcus Culture ID Test (Gen-Probe, San Diego, CA, USA).

### Antibiotic Susceptibility of Human Isolates

The antibiotic susceptibility profiles were studied with the disk diffusion method (patients 1 and 3) or Etest (patient 2). Results were interpreted according to the EUCAST rules (www.eucast.org/eucast_disk_diffusion_test/breakpoints/).

### Collection and Microbiological Characterization of Equine Isolates

None of the horses from the stables associated with the first 2 human cases (patients 1 and 2) showed any signs of respiratory illness. The horses from the third stable (owned by patient 3) were not examined; however, the owner did not recall any clinical signs of respiratory or other disease in his horses. Nasal swab specimens were collected from 7 horses owned by patient 1 (stable A) and 4 horses owned by patient 2 (stable H). The swabs were streaked onto bovine blood agar plates and incubated in a 5% CO_2_ atmosphere at 37°C (according to the standard operating procedures of the Finnish Food Safety Authority Evira, Kuopio, Finland) for 24 hours. β-hemolytic colonies were studied with conventional methods, and biochemical characterization was performed by using Rapid ID32 Strep (bioMérieux). *S. zooepidemicus* was isolated from 5 horses in stable A, but not from any horse in stable H. Six other *S. zooepidemicus* isolates from horses unrelated to the described human cases (stables B to F) (Table 2) were included in the genetic comparison.

**Table 2:** Molecular characterization of *Streptococcus equi* subspecies *zooepidemicus* isolates by sequencing of the SzP protein gene and by multilocus sequence typing*

Isolate ID no.	Origin	Year	Stable	MLST sequence type	SzP type	SzP GenBank accession no.
Hum1	Patient 1, blood	2011	A	ST-10	I	AF519489
Hum2	Patient 2, blood	2011	H	ST-10	I	AF519489
Hum3	Patient 3, aortic wall	2011	Not done	ST-209	VII	AF519488
642/11	Horse, nasal swab, nonclinical	2011	A	ST-147	IV	AF519482
645/11	Horse, nasal swab, nonclinical	2011	A	ST-175	II	AFKC287220†
646/11	Horse, nasal swab, nonclinical	2011	A	ST-66	V	AFKC287221†
647/11	Horse, nasal swab, nonclinical	2011	A	ST-175	II	AFKC287220†
648/11	Horse, nasal swab, nonclinical	2011	A	ST-10	I	AF519489
744/11	Horse, nasal swab, nonclinical‡	2011	C	ST-80	VIII	U04620
1128/11	Horse, foal, sepsis	2011	B	ST-5	VI	AFKC287222†
627/11	Horse, nasal swab, nonclinical‡	2011	C	ST-115	III	AF519478
6939/10	Horse, nasal swab, nonclinical‡	2010	D	ST-201	VII	AF519488
8110/09	Horse, synovial fluid (arthritis)	2009	E	ST-299†	III	AF519478
7723/09	Horse, foal, tracheal fluid, respiratory infection§	2009	F	ST-XX†¶	III	AF519478

*ID, identification; MLST, multilocus sequence typing. †Not previously described. ‡The samples were collected for screening of *S. equi* subsp. *equi,* but *S. equi* subsp. *zooepidemicus* was identified. §Co-infection with *Pasteurella* sp. and *S. suis.* ¶Recorded in the PubMLST database: 8 (arcC)–52 (nrdE)–2 (proS)–14 (spi)–1 (tdk)–22 (tpi)–n/a (*yqiL*).

### Pulsed-field Gel Electrophoresis

Three human isolates (1 from each patient) and 11 equine isolates of *S. zooepidemicus* were investigated by PFGE, sequencing of the *szP* gene, and MLST. DNA isolation was performed as described by Elliot et al. (*33*), and 40 U of *Sma*I was used for digestion. The chromosomal digests were separated by PFGE, with a switch time of 5 to 40 s for 20 h at a 120° angle and a voltage gradient of 6 V/cm at 12°C. Chromosomal DNA of *Salmonella enterica* serovar Braenderup H9182 was used as a marker.

### Sequencing of the *szP* gene and MLST

Isolates of *S. zooepidemicus* were cultured on 5% horse blood agar (National Veterinary Institute, Uppsala, Sweden) in a 5% CO_2_ atmosphere at 37°C for 24 h. Preparation of DNA from bacterial culture was performed by a boiling procedure; a 1-μL loop of bacteria was suspended in 100 μL of sterile H_2_O and incubated at 98°C for 15 min. The samples were centrifuged and the supernatants were collected and used as templates in the sequencing analyses.

The isolates of *S. zooepidemicus* (n = 14) were investigated by sequencing a 373-bp fragment of the SzP protein gene (*25*). Sequencing was performed according to Båverud et al. (*34*). Sequences were edited, assembled, and analyzed by using BioNumerics 6.5 (Applied Maths, Saint-Martens-Latem, Belgium).

MLST was performed according to Webb et al. (*22*). Sequences were edited, assembled, and analyzed by using BioNumerics 6.5. Sequence types (STs) were determined using the PubMLST *S. zooepidemicus* database.

## Results

### Microbiological Identification and Antibiotic Susceptibility of Human Isolates

The colonies of *S. zooepidemicus* on blood agar were large and mucoid and had a wide zone of β-hemolysis. All isolates were sensitive to erythromycin, clindamycin, penicillin, vancomycin, and cephalexin (data not shown). Microbiological identification data for the *S. zooepidemicus* isolates from human cases are shown in Table 1.

### Molecular Characterization of Isolates

**Figure 1:**
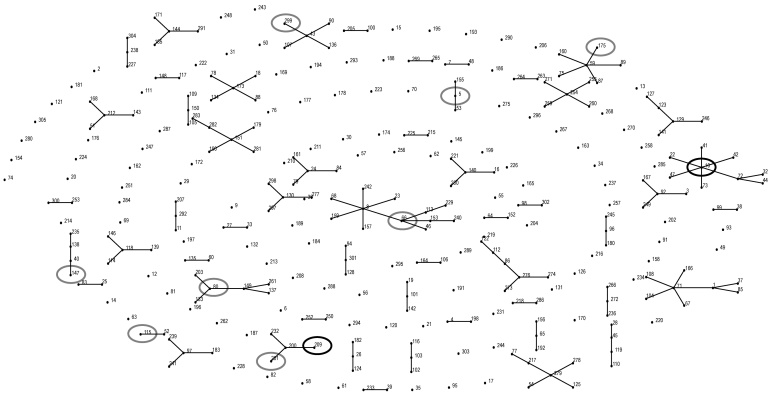
eBURST diagram (http://eburst.mlst.net) of all multilocus sequence typing (MLST) sequence types (STs) for *Streptococcus equi* subspecies *zooepidemicus* and *S. equi subsp. equi* recorded in the PubMLST database (http://pubmlst.org/szooepidemicus) (February 7, 2013). Single-locus variants (SLVs) are connected by a solid line. Black circles indicate strains isolated in this study from human cases and 1 horse (ST-10: Hum1, horse isolate 648/11, and Hum2; ST-209: Hum3 isolate). Gray circles indicate strains isolated from horses in this study. SzP protein sequence types were identical (GenBank accession no. AF519488) for the double-locus variants ST-201 and ST-209, whereas the SzP protein STs differed for strains not closely related by MLST (as described in Table 2).

The *S. zooepidemicus* isolates displayed 10 ST types by MLST. Their relatedness was compared by using eBurst (http://eburst.mlst.net) of all MLST STs for *S. equi* subsp. *zooepidemicus* and *S. equi* subsp. *equi* recorded in the PubMLST *S. zooepedemicus* database (February 7, 2013) (Figure 1). eBurst analysis indicated that ST-10, displayed by 3 isolates, Hum1, Hum2, and equine 648/11, was not related to any other STs of the *S. zooepidemicus* isolates examined in this study (Figure 1; Table 2). ST-209 and ST-201 are double-locus variants (DLVs) and were displayed by Hum3 isolate and horse isolate 6939/10, respectively. All other detected STs were unrelated to each other. Isolates from stables E (8110/09) and F (7723/09) displayed STs not previously described in the PubMLST *S. zooepedemicus* database. In addition, no product was obtained from forward and reverse primers for the *yqiL* gene from the isolate from stable F (7723/09).

**Figure 2:**
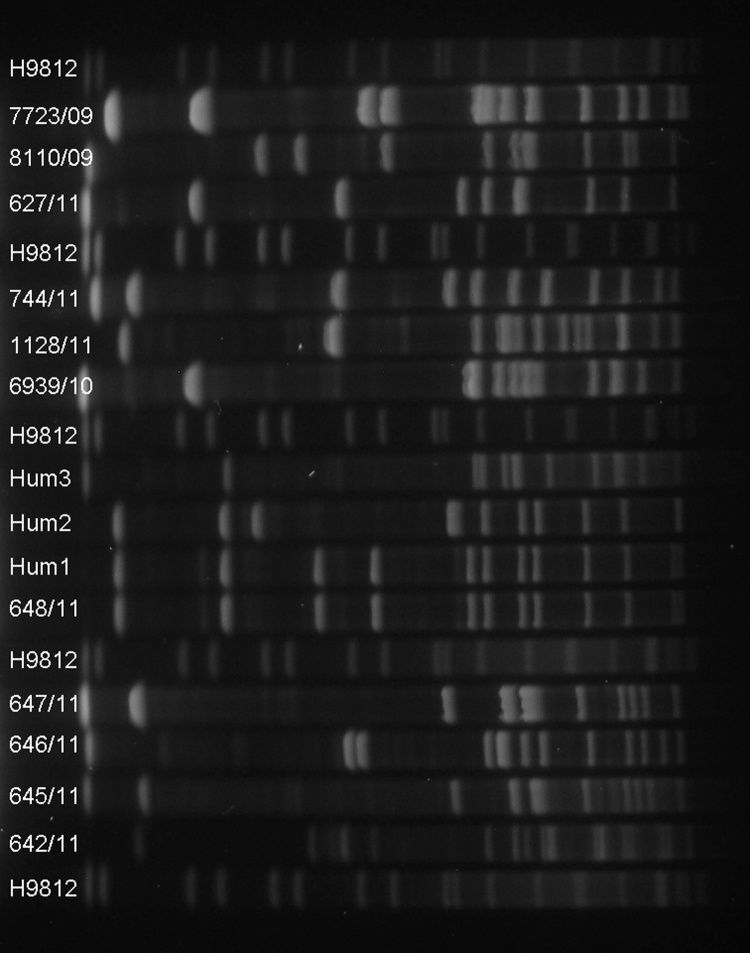
Pulsed-field gel electrophoresis of *Streptococcus equi* subspecies *zooepidemicus* isolates using *Sma*I. The lanes are marked with the number of the respective isolate. DNA of *Salmonella enterica* serovar Braenderup H9182 was used as a molecular marker.

The human isolates Hum1 (patient 1) and Hum2 (patient 2) displayed an *szP* sequence (GenBank accession no. AF519489) and MLST sequence type (ST-10) identical to the equine isolate 648/11 (stable A) (Table 2). Hum1 was also identical to equine isolate 648/11 on PFGE (Figure 2). Hum2, however, differed from Hum1 and 648/11 by 6 bands on the PFGE profile. The third human isolate, Hum3 (patient 3), was closely related to 1 equine isolate (6939/10) from an unrelated stable (stable D). These isolates displayed an identical *szP* sequence (accession no. AF519488). Their PFGE profiles were almost identical, and the MLST types ST-209 (Hum3) and ST-201 (6939/10) were DLVs. None of the other equine isolates displayed the same *szP* sequence type or MLST STs as the human isolates. Among the 5 *S. zooepidemicus* isolates from stable A, 645/11 was identical to 647/11 on the basis of the MLST ST (ST-175), *szP* type (II), and PFGE profile. All other isolates differed from each other. Several equine isolates displayed *szP* sequences not previously described in GenBank (645/11, 646/11, 647/11, and 1128). All *szP* sequencing results and corresponding GenBank accession numbers are listed in Table 2. 

## Discussion

We report 3 unrelated cases of *S. zooepidemicus* infection in patients from eastern Finland who had close and continuous contact with horses. It is noteworthy that the disease in all 3 patients was invasive and severe, requiring prolonged treatment and rehabilitation. Sepsis occurred in 2 cases (patients 1 and 2), meningitis and endocarditis in 1 (patient 1), purulent arthritis in 1 (patient 2), and a psoas abscess in connection with an aortic wall infection in 1 (patient 3). In patient 3, transient bacteremia might have occurred earlier.

MLST, PFGE, and sequencing of the SzP protein gene demonstrated identical profiles in a human isolate (Hum1) with an equine isolate (648/11), which strongly supports the zoonotic nature of this disease. Notably, the strain (ST-10) colonized the horse’s nostrils and acted as an innocent commensal, whereas in humans this strain appeared highly virulent and caused severe illness. In the second case (patient 2), we were unable to isolate the same strain from his horses. This failure may have been due to a transient *S. zooepidemicus* carriage in the nasopharynx, lymphoid tissues, or respiratory tract of the horse. Patient 2 might have been in contact with other horses as well. The strains from patient 1 and patient 2 were identical according to both *szP* sequencing and MLST, which supports the close relationship between the Hum1 and Hum2 isolates, and although the 2 isolates differed on PFGE analysis (Figure 1), the data strongly suggest that the infection of patient 2 was also transmitted zoonotically. ST-10 is a single-locus variant (SLV) of ST-72, which previously has been isolated from a case of human nephritis in the UK in 1983 (http://pubmlst.org/szooepidemicus/), and from a large outbreak of severe human nephritis in Brazil during 1997 and 1998 associated with consumption of unpasteurized cheese (*13**,**35**,**36*). The isolated strain in the Brazil outbreak was shown to have several genetic similarities to group A streptococci (*35*).

PFGE reveals random genetic events, such as point mutations or insertions or deletions of genetic material (*37*), thereby being often a more sensitive method than MLST to identify recent epidemic strains. However, it is not possible to estimate whether *S. zooepidemicus* isolates from patients 1 and 2, with altered PFGE profiles and approximately a 3-months’ gap between the diagnoses of disease, could be of the same origin. Notably, the strain isolated from patient 3 (Hum3), which differed completely from Hum1 and Hum2, was identical by MLST (ST-209) to a strain isolated from horses in an outbreak of respiratory disease in Iceland in 2010 (Bjornsdottir et al., unpub data). The Hum3 isolate also shared a *szP* sequence type (accession no. AF519488) previously found in horses with respiratory disease (S.B. Lindahl, unpub. data) as well as in an asymptomatic horse (6939/10) in this study. The ST-209 strain has further been isolated from a person with septicemia (that was associated with abortion) in Iceland in 2010, and has a SLV (ST-200) and a DLV (ST-201) that have been reported from cases of abortion/uterine infections in horses (http://pubmlst.org/szooepidemicus/). The ST-201 was also found in one of the healthy horses in this study (Table 2). 

All strains of *S. zooepidemicus* displayed mucoid colonies on the agar plates, indicating expression of a hyaluronic acid capsule, a well-known virulence factor in other pathogenic streptococci, such as *S. equi* in horses and *S. pyogenes* in humans. However, the expression of the mucoid capsule was variable: Hum1 strain produced large and highly mucous colonies, whereas those from Hum3 were heterogeneous in colony size and less mucoid. Whether there is a correlation between the production of mucinous substance and severity of the disease remains to be determined. Additional virulence factors, such as the presence of sAgs (*16*), would be intriguing to investigate. In *S. pyogenes*, variation in the M-protein is attributed to variable virulence. For example, the M1 strains are the most pathogenic (*14*). The sequence variants of the SzP protein gene in *S. zooepidemicus* were investigated but could not be correlated with clinical features in horses in a study by Walker and Runyan (*26*). However, determining such a correlation might be possible for the human isolates.

Recently, an outbreak of invasive *S. zooepidemicus* infection has been reported from Finland by Kuusi et al. (*30*). Altogether, 7 patients were identified: 6 had septicemia and 1 had purulent arthritis. All had consumed goat cheese produced from unpasteurized milk in a small-scale dairy. In Finland (population 5.2 million), all invasive streptococcal infections must be reported to the National Infectious Disease Register. As reviewed by Kuusi et al., only 3 cases of invasive *S. zooepidemicus* infections were reported to the register from 1992 through 2002, and ≈10 cases of invasive group C streptococcal infections occurred annually. In other words, even invasive isolates were often typed only to the Lancefield group level.

The novelty of our investigation is that an identical *S. zooepidemicus* strain was isolated from patient 1 and from a healthy horse in his stable, suggesting zoonotic transmission. Furthermore, patient 2 was infected with a *S. zooepidemicus* strain clonally related to that of patient 1, as judged by 2 independent typing methods, although patients 1 and 2 lived 140 km apart without a verified contact with each other. Notably, the isolate from patient 1 was highly virulent in humans but did not cause any clinical infection in the horse. In contrast, the isolate from patient 3 had the same MLST type as the strain previously isolated from several horses in an outbreak of respiratory disease. Our work yielded 3 new sequences of the *szP* gene, deposited under GenBank accession nos. KC287220 (isolate 645/11), KC287221 (isolate 646/11), and KC287222 (isolate 1128/11). Further, isolate 8110/09 was added to the PubMLST *S. zooepidemicus* database with ST-299. The isolate 7723/09 could not be assigned a ST because there was no product for the *yqiL* gene; however, the isolate is recorded in the PubMLST *S. zooepidemicus* database with the following allele sequence: 8 (arcC)–52 (nrdE)–2 (proS)–14 (spi)–1 (tdk)–22 (tpi)–n/a (*yqiL*). 

## Conclusion

Leisure and professional equine sports activities are growing in many countries. *S. zooepidemicus* infection transmitted from horses may cause severe illness in humans and should be considered an emerging zoonosis. Bacteriological identification of *S. zooepidemicus* is cheap and feasible with simple fermentation methods. Therefore, typing to the species level is strongly recommended for all clinical laboratories whenever group C streptococci are recovered from severely infected persons. Early identification of *S. zooepidemicus* will facilitate appropriate medical intervention and timely epidemiologic surveillance and finally, prevent the spread of a potentially life-threatening pathogen.
